# Seed development-related genes contribute to high yield heterosis in integrated utilization of elite autotetraploid and neo-tetraploid rice

**DOI:** 10.3389/fpls.2024.1421207

**Published:** 2024-06-12

**Authors:** Zijun Lu, Weicong Huang, Qi Ge, Guobin Liang, Lixia Sun, Jinwen Wu, Fozia Ghouri, Muhammad Qasim Shahid, Xiangdong Liu

**Affiliations:** ^1^ State Key Laboratory for Conservation and Utilization of Subtropical Agro-Bioresources, Guangdong Laboratory for Lingnan Modern Agriculture, South China Agricultural University, Guangzhou, China; ^2^ School of Biology and Agriculture, Shaoguan University, Shaoguan, China; ^3^ Guangdong Provincial Key Laboratory of Plant Molecular Breeding, South China Agricultural University, Guangzhou, China; ^4^ Guangdong Base Bank for Lingnan Rice Germplasm Resources, College of Agriculture, South China Agricultural University, Guangzhou, China

**Keywords:** *Indica* autotetraploid rice, neo-tetraploid rice, heterosis, grain weight, grain yield

## Abstract

**Introduction:**

Autotetraploid rice holds high resistance to abiotic stress and substantial promise for yield increase, but it could not be commercially used because of low fertility. Thus, our team developed neo-tetraploid rice with high fertility and hybrid vigor when crossed with *indica* autotetraploid rice. Despite these advances, the molecular mechanisms underlying this heterosis remain poorly understood.

**Methods:**

An elite *indica* autotetraploid rice line (HD11) was used to cross with neo-tetraploid rice, and 34 hybrids were obtained to evaluate agronomic traits related to yield. WE-CLSM, RNA-seq, and CRISPR/Cas9 were employed to observe endosperm structure and identify candidate genes from two represent hybrids.

**Results and discussion:**

These hybrids showed high seed setting and an approximately 55% increase in 1000-grain weight, some of which achieved grain yields comparable to those of the diploid rice variety. The endosperm observations indicated that the starch grains in the hybrids were more compact than those in paternal lines. A total of 119 seed heterosis related genes (SHRGs) with different expressions were identified, which might contribute to high 1000-grain weight heterosis in neo-tetraploid hybrids. Among them, 12 genes had been found to regulate grain weight formation, including *OsFl3, ONAC023, OsNAC024, ONAC025, ONAC026, RAG2, FLO4, FLO11, OsISA1, OsNF-YB1, NF-YC12*, and *OsYUC9*. Haplotype analyses of these 12 genes revealed the various effects on grain weight among different haplotypes. The hybrids could polymerize more dominant haplotypes of above grain weight regulators than any homozygous cultivar. Moreover, two SHRGs (*OsFl3* and *SHRG2*) mutants displayed a significant reduction in 1000-grain weight and an increase in grain chalkiness, indicating that *OsFl3* and *SHRG2* positively regulate grain weight. Our research has identified a valuable *indica* autotetraploid germplasm for generating strong yield heterosis in combination with neo-tetraploid lines and gaining molecular insights into the regulatory processes of heterosis in tetraploid rice.

## Introduction

Polyploidy is widespread in plants, including 30~50% flowering plants, 50~70% angiosperms, and many important crops like wheat, potato, sugarcane, cotton, and rape seed (Comai, 2005; Alix et al., 2017). Polyploid individuals possess more than two homologous chromosomes, which can be categorized as autopolyploidy and allopolyploidy based on the origin of the additional chromosomes. The chromosomes of the autopolyploidy originate from the same species, like autotetraploid rice (AAAA genome), while the chromosomes in allopolyploidy originate from different species, such as allohexaploid wheat (AABBDD genome) (International Wheat Genome Sequencing Consortium, 2018). Polyploid plants exhibit robust growth, enhanced stress resistance, increased biosynthesis, improved nutrient composition, and stronger adaptability in plant evolution (Xu et al., 2011; Mclntyre, 2012; Allario et al., 2013; Chao et al., 2013; Corneillie et al., 2019; Wang et al., 2022).

Autotetraploid rice (ATR) is a useful germplasm developed from genome duplication of diploid rice, in which intersubspecific hybrids showed great biological vigor and high yield potential (Koide et al., 2020). However, the limited reproductive capacity of autotetraploid rice and its hybrids has impeded their widespread commercial cultivation (Wu et al., 2015). Prior studies have indicated that autotetraploid sterility may be attributed to irregular meiotic chromosomal behaviors, changes in DNA methylation, and disrupted gene or non-coding RNA expression (He et al., 2011a; Wu et al., 2014, 2015; Li et al., 2016b, 2018, 2020). To dissolve this “bottleneck” problem (polyploidization sterility), Chinese scientists developed some tetraploid rice with high fertility by many year’s effort, including PMeS polyploid rice and neo-tetraploid rice (NTR, 80% seed setting) (He et al., 2011b; Guo et al., 2017; Ghaleb et al., 2020; Liu et al., 2023). NTR lines had the ability to overcome the polyploidization sterility when they crossed with typical autotetraploid rice with low fertility (Guo et al., 2017; Ghaleb et al., 2020; Yu et al., 2020). NTR lines were clustered into one independent group adjacent to the *japonica* subspecies in a comparative genomic study (Yu et al., 2021). On the other hand, NTR lines harbored wide compatibility gene *S_5_^n^* and pollen fertility “neutral gene” *Sc^n^* (Ghaleb et al., 2020). Thus, those hybrids derived from NTR and *indica* autotetraploid lines demonstrated no hybrid sterility and significant yield heterosis (Guo et al., 2017; Chen et al., 2019; Ghaleb et al., 2020), indicating that NTR can serve as the primary parental lines for restorer lines in future intersubspecific tetraploid hybrid breeding.

In the past 20 years, our group developed more than 100 ATR lines. The highlight one of these lines, HD11, was derived from progenies resulting from the self-pollination of Huanghuazhan-4x (HHZ-4x), whose hybrids showed significant heterosis and good plant performance. In this study, HD11, 34 NTR lines, and their hybrids were developed to evaluate intersubspecific tetraploid heterosis, two of which were used to ascertain the genes associated with the production of heterosis in grain weight. Our study aims to provide a yield improvement case of polyploid rice by utilizing superior genetic resources and offer a distinct perspective on understanding the mechanisms behind heterosis regulation.

## Materials and methods

### Plant materials

The autotetraploid rice, HD11, was developed from the 8^th^ generation of self-pollination of Huanghuazhan-4x (HHZ-4x). HHZ-4x was developed from genome duplication of the diploid cultivar Huanghuazhan (*Oryza sativa* L. ssp. *indica*) by colchicine treatment in our lab. Two neo-tetraploid lines with high fertility, Huaduo1 (H1) and Huaduo8 (H8), were used as paternal lines of two tetraploid hybrids, 1HF_1_ and 8HF_1_. Moreover, 34 hybrids were developed using HD11 by crossing 34 neo-tetraploid rice lines. The *OsFl3* and *SHRG2* mutants were genetically modified in the ZH11 background using the CRISPR/Cas9 system.

### Investigation of agronomic traits and evaluation of heterosis

Yield-related traits, such as panicle number, total grain number, seed setting rate, 1000-grain weight, and grain yield per plant, were investigated. The high-parent heterosis was calculated as described by Guo et al. (2017): HPH = (F_1_-HP)/HP×100%; F_1_ indicates the performance of hybrid plants; HP signifies superior performance in both parents.

### Whole-mount eosin B-staining confocal laser scanning microscopy observations

To characterize the endosperm structure of mature seeds, WE-CLSM observations were performed as follows: The brown rice was cut by a sharp blade and stained by 4% eosin B solution for 5 min, hyalinized via pure methyl salicylate before observation under WE-CLSM. WE-CLSM observations were also performed to characterize the endosperm and embryo development in 5P ovaries, as described in our previous study (Li et al., 2023). The collected samples were fixed in FAA solution (70% ethanol: acetic acid: methanal = 89:5:5, v/v), went through gradient rehydration, stained by 4% eosin B solution, dehydrated by gradient ethanol, and hyalinized via 50% and pure methyl salicylate before observation under WE-CLSM.

### RNA-seq analysis

A total of 30 samples were collected and stored at -80°C. These samples consisted of two developmental stages of the ovary (flowering stage, 0P; 5 days after pollination, 5P) from five different materials [HD11, H1, F_1_(HD11×H1), H8, F_1_(HD11×H8)]. Each sample was collected in three biological replications. The total RNA extraction and RNA-seq were done as described by Guo et al. (2017). Trimmomatic software was used to filter low-quality data (Bolger et al., 2014). STAR and samtools were used to map reads to MSU7.0 Nipponbare reference genome and evaluate the expression level (FPKM values) of genes (Li et al., 2009; Dobin et al., 2013). The differentially expressed genes (DEG) were identified according to the following criterion: (1) |log_2_(fold change)| >1; (2) *P*-value <0.05 (False discovery rate control method); (3) At least one sample with FPKM>10.

### Bioinformatics tools

Those candidate genes are annotated in the National Rice Data Center website (Kawahara et al., 2013). The global gene expression profile of target genes was predicted by using the Rice eFP expression profile analysis website (Winter et al., 2007). Venn analyses, upset plot analyses and heatmap diagrams were performed by TBtools (Chen et al., 2023). Haplotype analyses were performed via RFGB v2.0 tools (Wang et al., 2018; Wang et al., 2020a).

### Identification of CRISPR/Cas9 mutants

Single target targeting coding sequences of *OsFl3* (5’-GCACTAGCCATCACAAC-3’) or *SHRG2* (5’-ACATATCTTGTTCTAGT-3’) were designed for CRISPR/Cas9 system to obtain transgenic lines. All transgenic seedlings naturally grew at the experimental station of South China Agricultural University, Guangzhou, China. The targeted sites of *OsFl3* and *SHRG2* were amplified from transgenic plants for Sanger sequencing to select homozygous mutations. The PCR primers were designed by Primer Premier 5.0 (**Supplementary Table S1**).

## Results

### Production assessment of an *indica* autotetraploid line, HD11, and its intersubspecific hybrids

**Figure 1:**
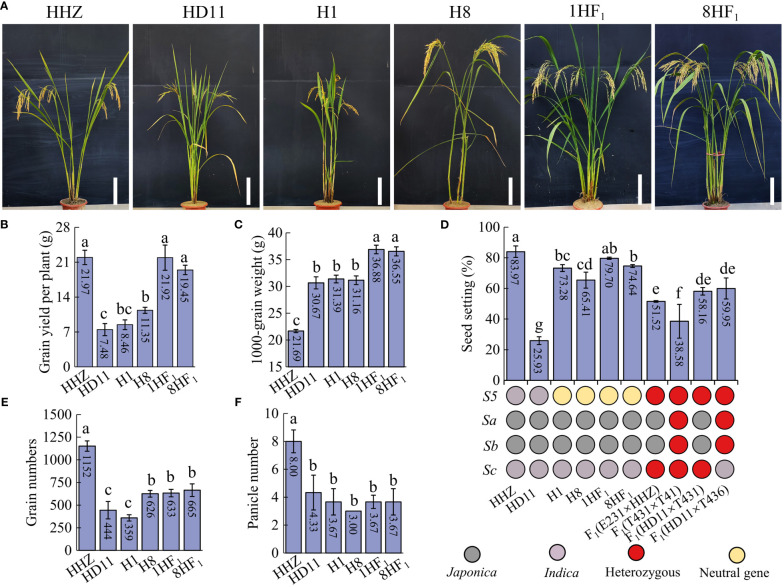
Yield-related traits evaluation of intersubspecific tetraploid hybrids and parental lines. **(A)** Plant morphology, **(B)** grain yield per plant, **(C)** 1000-grain weight, **(D)** seed setting rate, **(E)** grain numbers, and **(F)** panicle number of six lines. *S_5_*, *Sa*, *Sb*, and *Sc* indicate intersubspecific hybrid sterile loci. 1HF_1_ and 8HF_1_ indicate F_1_(HD11×H1) and F_1_(HD11×H8). Error bars indicate the standard deviation (SD) with *n* = 3. Significant differences were indicated by different lowercase letters (one-way ANOVA, least significant difference (LSD) test, *P* < 0.05).

In order to create superior *indica* ATR cultivars, we produced a set of *indica* ATR lines using high-yielding *indica* diploid lines. Among these lines, we isolated an ATR line called HD11 in 2020. HD11 exhibited outstanding plant performance and was obtained from progenies of HHZ-4x by our research group. Interestingly, when HD11 was crossed with NTR lines, the resulting hybrids exhibited a substantial increase in yield. A total of 34 hybrids developed from HD11 crossing with NTR lines displayed high plant yield, seed setting and 1000-grain weight with high-parent heterosis, indicating the strong combination between HD11 and NTR lines (**Supplementary Table S2**). Among them, two hybrids, 1HF_1_ [F_1_(HD11×H1)] (**Supplementary Figure S1**) and 8HF_1_ [F_1_(HD11×H8)], displayed 159.10% and 71.37% high-parent yield heterosis (**Figure 1A**), respectively, which were commensurate to the yield of the commercial rice variety (HHZ) (**Figure 1B**). Moreover, 1HF_1_ and 8HF_1_ exhibited 17.49% and 17.30% high-parent heterosis in 1000-grain weight, respectively. In this case, 1000-grain weight of 1HF_1_ (36.88 g) and 8HF_1_ (36.55 g) showed a 68.51~70.03% increase compared to HHZ (21.69 g) (**Figure 1C**). For seed setting, 1HF_1_ (79.70%) and 8HF_1_ (74.64%) were normal fertile and significantly higher than those hybrids of ATR lines, such as HD11×T431*^japonica^* (58.16%), HD11×T436*^japonica^* (59.95%), and T431×T41*^indica^* (38.58%) (**Figure 1D**). For grain number per plant, 1HF_1_ (632.67) and 8HF_1_ (665.33) were still significantly lower than HHZ (1152.00), but significantly increased relative to HD11 (444.00) and H1 (359.33) (**Figure 1E**). For panicle number, no obvious improvement was found in 1HF_1_ (3.67) and 8HF_1_ (3.67) relative to parental lines, HD11 (4.33), H1 (3.67) and H8 (3.00), which is still significantly inferior to HHZ (8.00) (**Figure 1F**). Taken together, these findings demonstrated the potential for increasing crop productivity by combining the utilization strategy of polyploidization and intersubspecific heterosis.

Re-sequencing was employed to analyze the genomic DNA polymorphisms of HD11 compared with HHZ, 5 ATR lines, and 3 NTR lines. The evaluation of Q30 bases proportion, average depth, and coverage_10× showed that the quality of these resequencing data was high enough (Yu et al., 2021). A total of 1321 genes with specific variations were detected in HD11 compared to HHZ, out of which 28 are known to have a function (**Supplementary Table S3**). Gene ontology (GO) enrichment analysis identified 22 prominent terms in the biological process category associated with the mutant genes (**Supplementary Table S4**). A total of 14371 genes with specific variations were detected in HD11 compared to other ATR lines, of which 212 have known functions, including 60 resistance or tolerance-related genes and 54 physiological trait genes (**Supplementary Table S5**), which enriched in 14 Gene ontology (GO) biological process terms (**Supplementary Table S6**). A total of 8260 genes with distinct variations were found in HD11 compared with NTR, which were enriched in 16 prominent GO terms in the biological process category (**Supplementary Table S7**). Among those specific variant genes compared to NTR, 190 have known functions, including 45 physiological trait genes, 10 genes associated with yield components, 7 heading date genes, and 1 seed morphology gene (**Supplementary Table S8**).

### Grain weight formation among intersubspecific tetraploid hybrids and parental lines

**Figure 2:**
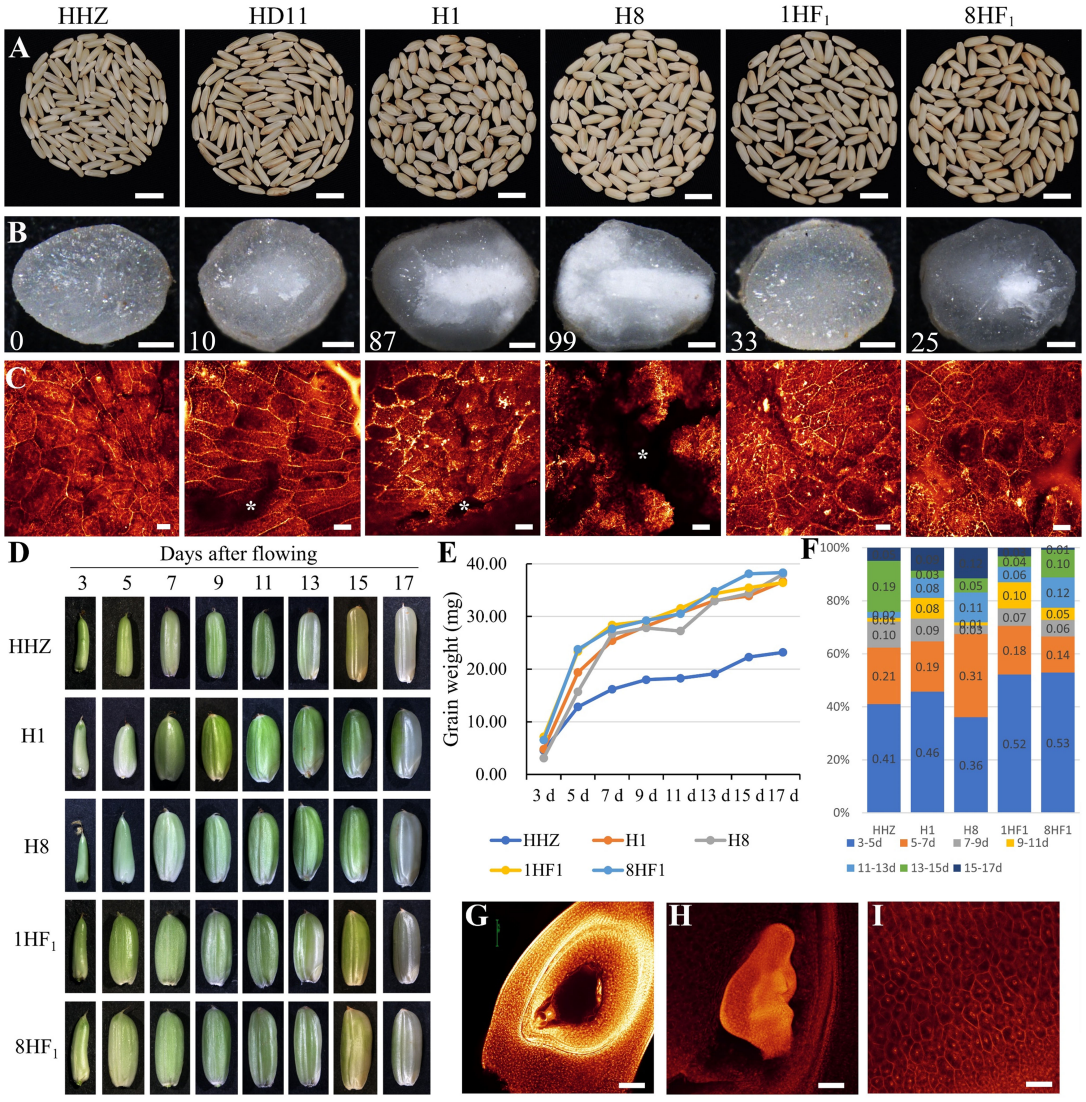
Growth analysis of developing caryopses of 1HF_1_, 8HF_1_, and parental lines. **(A, B)** Brown rice grains, **(C)** endosperm observation on the middle of grains (cross section) via WE-CLSM, **(D)** developing grains, **(E)** developing grain weight, **(F)** increase rate per 2 days of developing grains of HHZ, H1, H8, 1HF_1_, and 8HF_1_. **(G)** Embryo sac view before pollination (WE-CLSM). **(H)** Embryo and **(I)** endosperm at 5 days after pollination (WE-CLSM). **(B)** Numbers indicate the number of chalky grains per 100 grains. White “*” indicates unfilled interstices in the endosperm. Bars = 1 cm **(A)**, 50 µm **(B)**, 40 µm **(C, G-I)**.

Relative to diploid HHZ, the chalkiness increased in HD11 grains. Interestingly, the chalkiness in 1HF_1_ or 8HF_1_ grains was less than HD11, H1, and H8, suggesting that improved grain weight formation plays an important role in yield heterosis of 1HF_1_ and 8HF_1_ (**Figures 2A, B**). WE-CLSM observation confirmed denser starch grains in the 67.00~75.00% endosperms of hybrids, while severe interstices were observed in 87.00~99.00% endosperm of paternal lines (**Figure 2C**). We further characterized the grain development of HHZ, H1, H8, 1HF_1_, and 8HF_1_ to identify differences during grain weight heterosis formation. The developing grain weights of 1HF_1_ and 8HF_1_ were significantly higher than HHZ, H1, and H8 at 3 days after pollination. The increase of grain weight in 1HF_1_ and 8HF_1_ reached a plateau at 15 days after pollination, which was obviously earlier than H1 and H8 (**Figures 2D, E**). When we evaluate the increased grain weight per two days from 3 to 17 days after pollination, all HHZ, H1, H8, 1HF_1_, and 8HF_1_ increased 36~53% grain weight in the first two days, while 1HF_1_ and 8HF_1_ accumulated more grain weight (52~53%) than HHZ (41%), H1 (46%), and H8 (36%) (**Figure 2F**). WE-CLSM observation revealed detailed information about ovary development before and after fertilization. Before pollination, the egg cell, synergid, central cell and antipodal cells were observed in embryo sac (**Figure 2G**), while the embryo has been differentiated from the zygote, and the endosperm cells have filled the hole of the ovary at 5 days after pollination (**Figures 2H, I**). These results indicate that 5 days after pollination is an important stage for different grain weight among HHZ, H1, H8, 1HF_1_, and 8HF_1_.

### RNA-seq analyses detected the genes with higher expression level in tetraploid intersubspecific hybrids than parental lines

**Figure 3:**
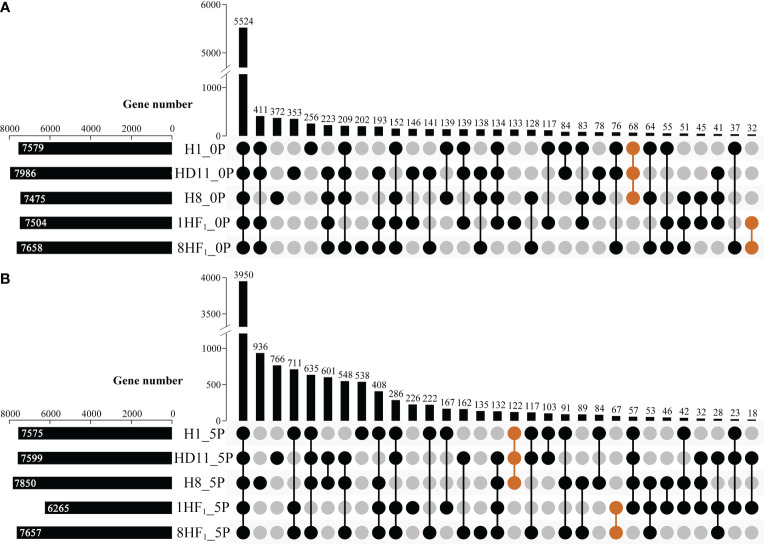
Upset plot analyses of expressed genes in tetraploid lines and their hybrids. **(A)** Upset plot analyses of expressed genes in 0P ovaries of tetraploid lines and their hybrids; **(B)** Upset plot analyses of expressed genes in 5P ovaries of tetraploid lines and their hybrids. Orange groups indicate that gene sets exhibit specificity in either parental lines or hybrids.

To reveal the genes related to strong heterosis formation during seed development of tetraploid intersubspecific hybrids, RNA-seq was performed to assess the global gene expression in developing seeds during two stages (0P, flowering; 5P, 5 days after pollination) among 1HF_1_, 8HF_1_ and three parental lines. More than 39.8 million clean reads were produced from each library, which could cover 91.85~96.08% of the reference genome (MSU7.0). While counting the number of genes expressed in each sample (FPKM>10), each material expressed a range of 7475 to 7986 genes in 0P seeds and a range of 6265 to 7850 genes in 5P seeds, respectively (**Figure 3**). Among them, 68 (0P) and 122 (5P) genes were expressed in three parental lines but not in two hybrids, while 32 (0P) and 67 (5P) genes were expressed in two hybrids but not in three parental lines (**Supplementary Table S9**). These specific genes might contribute to strong yield heterosis formation of tetraploid hybrids.

We analyzed the differentially expressed genes (DEGs) in 0P seeds. Relative to H1, 414 DEGs were identified in 1HF_1_ plants, including 156 up- and 258 down-regulated DEGs. Relative to HD11, 410 DEGs were identified in 1HF_1_ plants, including 136 up- and 274 down-regulated DEGs. Furthermore, there were 102 common DEGs (coDEGs) shared in 1HF_1_/H1 and 1HF_1_/HD11, including 13 up- and 89 down-regulated coDEGs (**Supplementary Figure S2A**; **Supplementary Table S10**). In our previous study, 819 DEGs (402 up- and 417 down-regulated) were identified in comparative analyses between 8HF_1_ and H8, and 592 DEGs (284 up- and 308 down-regulated) were identified in the comparative studies between 8HF_1_ and HD11. Furthermore, there were 86 coDEGs shared in 8HF_1_/H8 and 8HF_1_/HD11, including 32 up- and 54 down-regulated coDEGs (**Supplementary Figure S2B**; **Supplementary Table S10**).

Secondly, we analyzed the DEGs in 5P seeds. Relative to H1, 651 DEGs were identified in 1HF_1_ plants, including 314 up- and 337 down-regulated DEGs. Relative to HD11, 3996 DEGs were identified in 1HF_1_ plants, including 1972 up- and 2024 down-regulated DEGs. Furthermore, there were 341 common DEGs (coDEGs) shared in 1HF_1_/H1 and 1HF_1_/HD11, including 248 up- and 93 down-regulated coDEGs (**Supplementary Figure S2C**; **Supplementary Table S11**). In our previous study, 2235 DEGs (1151 up- and 1084 down-regulated) were identified in 8HF_1_/H8 comparative analyses, and 1737 DEGs (1229 up- and 508 down-) were identified in 8HF_1_/HD11 comparative analyses. Furthermore, there were 821 common DEGs (coDEGs) shared in 8HF_1_/H8 and 8HF_1_/HD11, including 722 up- and 99 down-regulated coDEGs (**Supplementary Figure S2D**; **Supplementary Table S12**). These coDEGs are hypothesized to have significant implications in the phenomenon of polyploid heterosis during seed development. In comparison to the 0P stage, a greater number of DEGs were identified during the 5P stage, suggesting that the 5P stage is an important period for grain weight heterosis.

Finally, we sought to identify genes with high-parent heterosis of expression level in both 1HF_1_ and 8HF_1_, which play a crucial role in facilitating robust yield heterosis in tetraploid hybrids. In 0P seeds, seven genes were identified, including 1 common up-regulated and 6 common down-regulated genes in both 1HF_1_ and 8HF_1_ (**Supplementary Figure S2E**). In 5P seeds, 112 genes were identified, including 107 common up-regulated and 5 common down-regulated genes in both 1HF_1_ and 8HF_1_ (**Supplementary Figure S2F**). These 119 candidate genes with high-parent heterosis in expression level were designed as seed heterosis related genes (hereafter referred to as SHRGs) (**Supplementary Table S13**).

### Hybrids can pyramid more elite haplotypes of 12 known SHRGs

**Figure 4:**
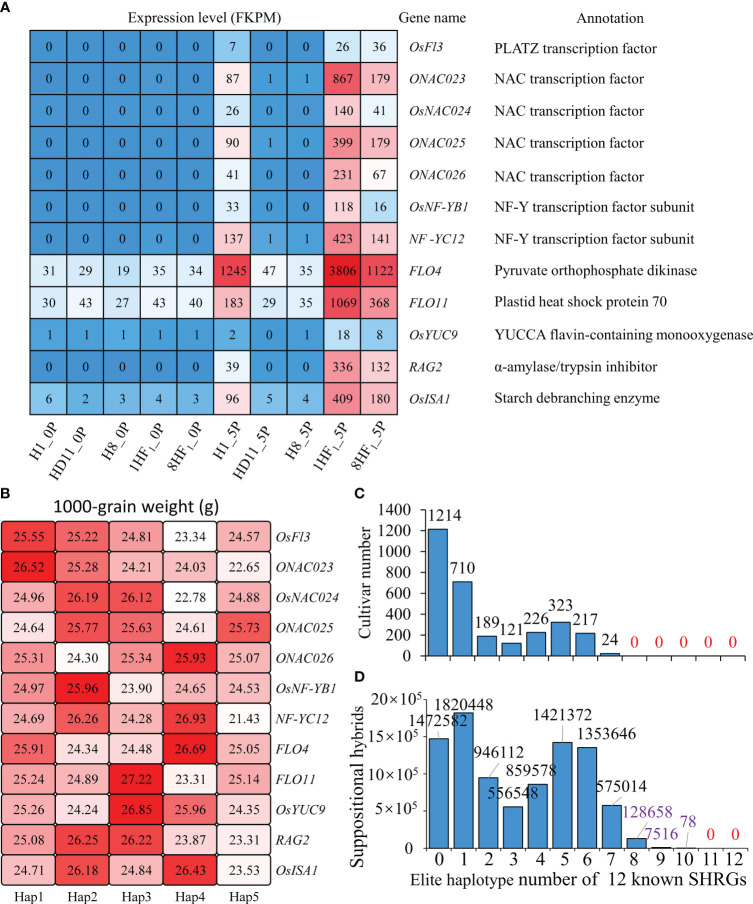
12 known grain weight regulated genes with higher expression levels in hybrids than parental lines. **(A)** Expression levels and functional annotation of 12 known SHRGs in 1HF_1_, 8HF_1_, and parental lines; **(B)** 1000-grain weight comparison among haplotypes of 12 known SHRGs; **(C)** Distribution of cultivars that carry different numbers of elite haplotypes in 12 known SHRGs; **(D)** Distribution of putative hybrids that carry different number of elite haplotypes in 12 known SHRGs.

A comprehensive analysis of 119 SHRGs revealed 12 identified positive regulators of grain weight, which functioned on endosperm development to impact both grain size and filling rate, including OsFl3 (PLATZ transcription factor), four NAC transcription factors (ONAC023, OsNAC024, ONAC025, and ONAC026), two subunits of NF-Y transcription factor (OsNF-YB1, NF-YC12), FLO4 (Pyruvate orthophosphate dikinase), FLO11 (Plastid heat shock protein 70), and OsYUC9 (YUCCA flavin-containing monooxygenase), RAG2 (α-amylase/trypsin inhibitor), and OsISA1 (Starch debranching enzyme). All of these 12 known SHRGs were up-regulated in hybrids (**Figure 4A**), which might contribute to an increase in grain weight and ultimately lead to high heterosis.

Haplotype analyses were conducted on the above-mentioned 12 known SHRGs using the RFGB database. While concentrating on their primary five haplotypes, the 1000-grain weight was compared among cultivars that possess distinct haplotypes of each known SHRG. Hap1-*OsFl3* (25.55 g), Hap1-*ONAC023* (26.52 g), Hap2-*OsNAC024* (26.19 g), Hap2-*ONAC025* (25.77 g), Hap4-*ONAC026* (25.93 g), Hap2-*OsNF-YB1* (25.96 g), Hap4-*NF-YC12* (26.93 g), Hap4-*FLO4* (26.69 g), Hap3-*FLO11* (27.22 g), Hap3-*OsYUC9* (26.85 g), Hap2-*RAG2* (26.25 g), Hap4-*OsISA1* (26.43 g) are the haplotypes with the highest 1000-grain weight (**Figure 4B**). If we suppose these haplotypes with the highest 1000-grain weight are the most elite haplotypes for each known SHRG to analyze the distribution of cultivars carrying different number of elite haplotypes, all 3024 cultivars have no more than 7 elite haplotypes for these 12 known SHRGs (**Figure 4C**). While randomly couple with two cultivars to construct suppositional hybrids and calculate their most elite haplotypes of 12 known SHRGs, 128658, 7516, and 78 hybrids could pyramid 8, 9 and 10 most elite haplotypes of 12 known SHRGs, respectively, which never exist in parental cultivars (**Figure 4D**). These findings indicate that the most superior genetic variations (haplotypes) are lacking in any rice cultivar. However, hybrids offer greater possibilities for pyramiding superior genetic variations of grain weight regulators and forming heterosis of grain weight.

### Functional verification of two selected SHRGs

**Figure 5:**
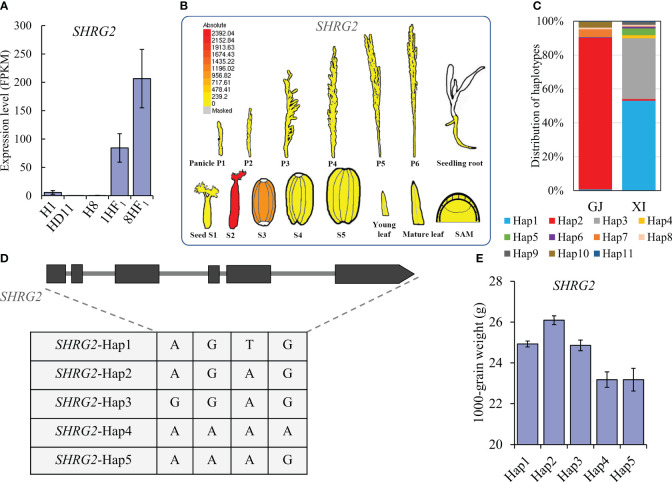
Haplotype and expression analyses of *SHRG2.* **(A)** Expression levels of *SHRG2* in 1HF_1_, 8HF_1_, and parental lines. **(B)** Expression pattern of *SHRG2* via eFP. **(C)** Haplotype distribution of *SHRG2* in *japonica* and *indica* cultivars of RFGB database. **(D)** Five main haplotypes of *SHRG2* in the cultivars of RFGB database. **(E)** 1000-grain weight comparison among cultivars with different *SHRG2* haplotypes.

To evaluate the biological relevance of 119 candidate SHRGs, we selected two SHRGs overlapped with 67 specific expressed genes in hybrids for functional verification in grain weight formation, *LOC_Os01g33350* (*OsFl3*) and *LOC_Os02g55210* (referred as *SHRG2*, here). Similar to *OsFl3*, *SHRG2* was a strongly expressed gene in both 1HF_1_ and 8HF_1_ 5P samples, which was almost completely suppressed in the 5P samples of the three parental lines (**Figures 4A**, **5A**). Expression pattern analyses via eFP tools revealed that *OsFl3* is mainly expressed in S3 developing seed, and *SHRG2* is primarily expressed in S2 developing seed (**Supplementary Figure S3**; **Figure 5B**). Haplotype analyses showed that haplotypes of *SHRG2* were distinguished between *indica* and *japonica* cultivars, which *japonica* cultivars mainly contained *SHRG2*-Hap2, and *indica* cultivars carried *SHRG2*-Hap1 or *SHRG2*-Hap3 (**Figures 5C, D**). The haplotypes of *SHRG2* affected 1000-grain weight in cultivars, while *SHRG2*-Hap2 showed the highest 1000-grain weight (**Figure 5E**).

**Figure 6:**
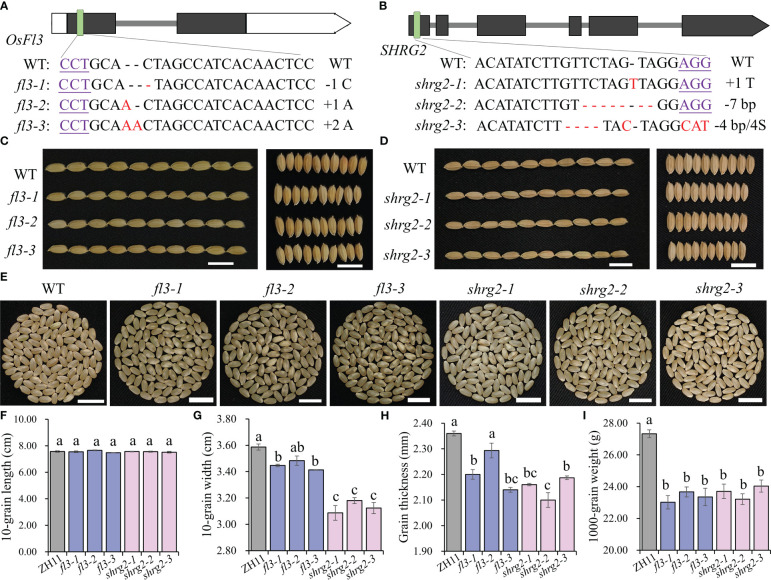
Functional verifications of *OsFl3* and *SHRG2*. **(A, B)** The schematic diagrams of *OsFl3* and *SHRG2* genes. The sequences of CRISPR/Cas9 target sites were given with protospacer adjacent motifs (PAMs) underlined and resulting mutations highlighted in red. The grain length and grain width of wild and mutant types **(C, D, F, G)**, brown rice grains **(E)**, grain thickness **(H)**, and 1000-grain weight **(I)** of ZH11, *fl3* and *shrg2*. Bars = 1 cm **(C–E)**. Different lowercase letters indicate significant differences (*P* < 0.05, one-way ANOVA, least significant difference (LSD) test). Error bars indicate the standard error (SE).

Moreover, we generated *fl3* and *shrg2* mutant in ZH11 background (*Oryza sativa* L. ssp. *Japonica*) via CRISPR/Cas9 system to verify grain weight regulated roles of *OsFl3* and *SHRG2*. Three homozygous lines with frameshift mutations were selected from *fl3* and *shrg2*, designated as *fl3-1*, *fl3-2*, *fl3-3*, *shrg2-1*, *shrg2-2*, and *shrg2-3*, respectively. The *fl3-1*, *fl3-2*, and *fl3-3* harbored a C deletion, an A insertion, and an AA insertion, while *shrg2-1*, *shrg2-2*, *shrg2-3* contained a T insertion, a 7 bp deletion, and a 4 bp deletion, respectively (**Figures 6A, B**). Mutation of *OsFl3* and *SHRG2* both caused a significant reduction in grain thickness and grain width and an increase in chalkiness, but did not affect grain length (**Figures 6C-H**). The 1000-grain weight of *fl3* (23.02~23.67 g) and *shrg2* (23.21~24.04 g) lines both displayed a significant reduction relative to ZH11 (27.33 g) (**Figure 6I**). Collectively, these findings indicate that *OsFl3* and *SHRG2* likely have significant functions in enhancing yield heterosis by controlling the development of grain weight.

## Discussion

### HD11 is an elite germplasm for improving the grain yield of neo-tetraploid hybrid rice

HD11 is a new autotetraploid line breed from the F_8_ self-crossing progenies of HHZ-4x. HHZ-4x is an autotetraploid rice developed from genome duplication of a diploid rice cultivar, HHZ (Huanghuazhan). HHZ is a highly esteemed diploid *indica* cultivar that exhibits semi-dwarfism, excellent eating quality, exceptionally high yield, and robust adaptation to diverse environments, and it has been cultivated across over 4.5 million ha in southern China (Zhou et al., 2016; Chen et al., 2017). HHZ contained 13.05% conserved regions and 86.95% recombined genome from a series of elite cultivars, such as Teqing (18.21%), Qingliuai (2.61%), Fengqingai (0.26%), Huangxinzhan (4.40%), Fenghuazhan (8.01%), Jingxian89 (0.74%), Texianzhan25 (0.65%), Huasizhan (8.62%), Fengbazhan (2.76%), Fengaizhan (0.62%), and Changsizhan (0.19%) (Zhou et al., 2016). HHZ carries a series of elite alleles regulating key agronomic traits, for example, *sd1*, *Ehd4*, *htd1*, *SSIIa*, *SSIIa*, *GS3*, *TAC1*, *SPK*, *RFT1*, *OsSSI*, *Amy3A*, *Gn1a*, *GW2*, *lp*, and *wx* (Qiu et al., 2018). HD11 contains 1321 specific mutant genes relative to HHZ, but larger differences were found between HD11 and ATR (14371 specific mutant genes) or NTR (8260 specific mutant genes), involving resistance or tolerance genes, physiological trait-related genes, and meiosis-related genes. The presence of these genetic variations is likely the primary factor that makes HD11 an excellent germplasm for crossbreeding with neo-tetraploid lines. This combination has shown promising results in enhancing the fertility and overall grain yields of intersubspecific tetraploid hybrid rice. The tetraploid hybrids in this study were able to attain equivalent grain yields to those of HHZ, because of overcoming the typically autotetraploid sterility, polyploidization advantages in increased 1000-grain weight, and intersubspecific heterosis in terms of 1000-grain weight, seed setting rate, and grain number.

### High-parent expressions of seed regulators contribute to grain weight heterosis in tetraploid rice

Many key heterosis-associated genes have been identified in rice, such as *Ghd7*, *Hd3a*, *Ghd8*, *TAC1*, *LAX1*, *OsMADS1*, *OsMADS51*, *GW6a*, *Hd1*, and *IPA1/OsSPL14*. Of these, *Ghd7*, *Hd3a*, *Ghd8*, *OsMADS51*, and *Hd1* regulates heading time (Kojima et al., 2002; Huang et al., 2016; Li et al., 2016a; Shao et al., 2019); *Ghd8* and *LAX1* regulate kernel number (Komatsu et al., 2001; Huang et al., 2016; Li et al., 2016a); *Ghd8* and *GW6a* regulate plant height (Sun et al., 2014; Huang et al., 2016; Li et al., 2016a); *IPA1* regulates plant architecture (Jiao et al., 2010; Miura et al., 2010); *OsMADS1* and *GW6a* regulate grain weight (Kim et al., 2007; Sun et al., 2014). In addition, RNA-seq analyses have identified numerous genes exhibiting distinct expression patterns in high-performing hybrid varieties such as Shanyou 63. These results suggest that the expression of heterosis-related genes may play a role in the establishment of yield heterosis (Guo et al., 2017; Chen et al., 2019; Shao et al., 2019; Ghaleb et al., 2020).

Here, we focused on the regulation of grain weight heterosis in intersubspecific tetraploid hybrids and used comparative RNA-seq analyses of developing ovary among intersubspecific autotetraploid hybrids and their parental lines to identify a key geneset (119 SHRGs) that might contribute to high grain weight heterosis in tetraploid hybrids. This geneset contains 13 explicit grain weight regulated genes, including *OsFl3*, *ONAC023*, *OsNAC024*, *ONAC025*, *ONAC026*, *RAG2*, *FLO4*, *FLO11*, *OsISA1*, *OsNF-YB1*, *NF-YC12*, *OsYUC9*, and *SHRG2*. Any mutation of *OsFl3* (Guo et al., 2022), *ONAC023* (Li et al., 2022), *RAG2* (Zhou et al., 2017), *FLO4* (Chastain et al., 2006), *FLO11* (Zhu et al., 2018; Tabassum et al., 2020), *OsISA1* (Chao et al., 2019), *OsNF-YB1* (Bai et al., 2016; Bello et al., 2019; Xu et al., 2021), *NF-YC12* (Bello et al., 2019; Xiong et al., 2019), *OsYUC9* (Xu et al., 2021), or double mutation of *OsNAC20* and *OsNAC26* (Wang et al., 2020b) would cause uncomplete grain filling and significantly increase chalkiness in seeds. Correspondingly, overexpression of *OsFl3* (Guo et al., 2022), *ONAC023* (Li et al., 2022), *RAG2* (Zhou et al., 2017), or *NF-YC12* (Xiong et al., 2019) would increase grain weight. *OsNAC024* and *ONAC025* contain SNPs that exhibit a noteworthy correlation with grain weight in rice, whose proteins interact with OsMED15a to govern the expression of grain weight genes, such as *GW2*, *GW5*, and *DR11* (Dwivedi et al., 2019). The *flo11* mutant exhibits temperature sensitivity in its phenotype (Tabassum et al., 2020). Sugar levels and its proteins influence the expression of OsNAC23 directly inhibit the transcription of *TPP1*, hence controlling sugar homeostasis and grain yield in rice (Li et al., 2022). Besides grain weight regulation, the NAC transcription factors, *OsNAC20* and *OsNAC26*, also positively regulate the expression of glutelin (*GluA1*/*B4/B5*), α-globulin and 16 kD prolamin (Wang et al., 2020b). In this study, phenotypic observations indicate that mutations in *OsFl3* or *SHRG2* lead to a decrease in grain weight due to impaired filling (**Figure 6**), further contributing to the understanding of 119 potential regulators of grain weight heterosis. These results suggest that all 13 grain weight regulators mentioned above function as positive regulators in the development of grain weight. The elite genotypes of these grain weight heterosis associate genes disperse in different varieties, while the generation of more elite genotypes in hybrids results in a higher expression level of grain weight regulators in the hybrids than their parental lines and promotes grain weight heterosis. Taken together, our study has presented a comprehensive analysis of the gene expression patterns in tetraploid rice, specifically focusing on the phenomenon of intersubspecific seed heterosis. We have found a set of genes that are associated with grain weight heterosis, thereby contributing to our understanding of the mechanisms underlying heterosis generation in neo-tetraploid rice.

### Breeding strategy for the utilization of multi-generation heterosis in neo-tetraploid rice

Our group also focused on the exploitation of those unique advantages of neo-tetraploid rice, such as multi-generation heterosis. Autotetraploid rice hybrids possess four homologous chromosomes, and their heterozygotes require more generations to become homozygous. As a result, these hybrids demonstrate robust heterosis for multiple generations. Previously, we had demonstrated that the hybrids of neo-tetraploid lines and *indica* autotetraploid lines exhibited near similar yield from F_2_ to F_4_ generation, indicating that the high levels of heterosis were maintained for several generations in the hybrids of neo-tetraploid rice crossed with autotetraploid rice (Chen et al., 2022). The multi-generation heterosis of tetraploid rice has great potential for producing hybrid seeds and reducing cost. In contrast to diploid rice, the key tetraploid progenitors must exhibit the capacity to overcome polyploid sterility, similar to our neo-tetraploid rice. Now, we have successfully bred a series of neo-tetraploid lines and identified an *indica* tetraploid germplasm, HD11, with a high combining ability to neo-tetraploid lines, which can work as *japonica* backbone parent and *indica* backbone parent in our future breeding, respectively. Thus, we proposed a strategy for utilizing multi-generation heterosis and intersubspecific heterosis based on neo-tetraploid rice (Chen et al., 2022; Liu et al., 2023). Referring to the “two-line” hybrid heterosis utilization in diploid rice involving a temperature-sensitive male sterile line (TMSL) and a restoring line (RL), our key strategy for future intersubspecific tetraploid hybrid rice breeding is as follows (**Supplementary Figure S4**):

(1) Creation of a new *indica* tetraploid TMSL with elite genes using HD11. The current study focuses on utilizing an exceptional tetraploid line, HD11, to enhance the crop productivity of tetraploid hybrid rice, which could be used for developing tetraploid TMSL. Previously, we confirmed the feasibility of creating tetraploid TMSL by editing the temperature-sensitive male sterile gene, *TMS5* (Chen et al., 2022). In this case, we can use CRISPR/Cas9 to target *TMS5* to develop HD11-drived TMSL.
(2) Breeding strong restorer lines based on neo-tetraploid rice. Neo-tetraploid rice can be used as the recurrent parent to cross with various autotetraploid lines, backcross 5-6 times assisted with molecular markers to select target genes (such as “wide compatibility genes” and “neutral genes” for pollen fertility), and finally self-cross to select excellent neo-tetraploid restorer lines. Robust restorers need to retain their capacity to overcome sterility caused by polyploidization, while also enhancing the quantity of grains and panicles.
(3) Selection of super vigor combinations of HD11-derived TMSL and neo-tetraploid restorer lines. HD11-drived TMSL can be used to cross with various neo-tetraploid restorer lines, and yield assessment of their F_1_ to F_4_ hybrids would be performed to identify super vigor combinations with high yield and multi-generation heterosis. Our group created several HD11-tms5 lines that were temperature-sensitive and identified several hybrids with high heterosis based on HD11 and neo-tetraploid restorer lines. Meanwhile, we also explore to “fix” the heterosis by apomixis using gene editing techniques. Additional efforts are needed in this aspect. It is important to acknowledge that there is significant room for genetic enhancement in tetraploid hybrids regarding grain quantity, panicle number, and tolerance to both biotic and abiotic stress. This implies a substantial potential for increasing grain yield. In order to get high yield, direct-seedling and dense planting could be tried in neo-tetraploid rice.

Furthermore, tetraploid rice possesses the distinctive benefit of multi-allelic heterosis, which can be effectively harnessed and applied in future breeding programs for tetraploid rice. Tetraploid hybrids can contain multiple alleles in the same locus, while only two alleles are possible in diploid hybrids. Our understanding of the phenomenon of additional heterosis in tetraploid hybrids with multiple alleles remains inadequate. Further investigation into the utilization of related traits is necessary for future tetraploid rice breeding.

## Conclusions

Yield assessment of intersubspecific autotetraploid hybrid rice offers empirical evidence for our tetraploid breeding strategy by the combination of elite *indica* autotetraploid lines and *japonica* neo-tetraploid lines. Intersubspecific autotetraploid hybrids still have excellent yield potential in the improvement of grain number, panicle number, elite haplotypes of grain weight regulators, and cultivation patterns. These results provide important germplasms for intersubspecific tetraploid hybrid rice breeding and new insights into the underlying mechanism of heterosis.

## Data availability statement

The raw reads of RNAseq were deposited in the NCBI Sequence Read Archive with accession ID PRJNA526117 (Samples of HD11, H8 and 8HF1), and NGDC BIG Submission with accession ID PRJCA023837 (Samples of H1 and 1HF1).

## Author contributions

ZL: Funding acquisition, Investigation, Software, Visualization, Writing – original draft, Writing – review & editing. WH: Formal analysis, Investigation, Methodology, Resources, Visualization, Writing – review & editing. QG: Investigation, Writing – review & editing. GL: Formal analysis, Methodology, Visualization, Writing – review & editing. LS: Investigation, Visualization, Writing – review & editing. JW: Validation, Writing – review & editing. FG: Investigation, Writing – review & editing. MS: Supervision, Writing – original draft, Writing – review & editing. XL: Conceptualization, Funding acquisition, Project administration, Supervision, Validation, Writing – original draft, Writing – review & editing.
