# Supplementary material for: Seed development-related genes contribute to high yield heterosis in integrated utilization of elite autotetraploid and neo-tetraploid rice
Source: Front Plant Sci. 2024 Jun 12;15:1421207. doi: 10.3389/fpls.2024.1421207 (PMC11204133; doi:10.3389/fpls.2024.1421207)
Supplement: Supplementary file 1 [file DataSheet_1.pdf]

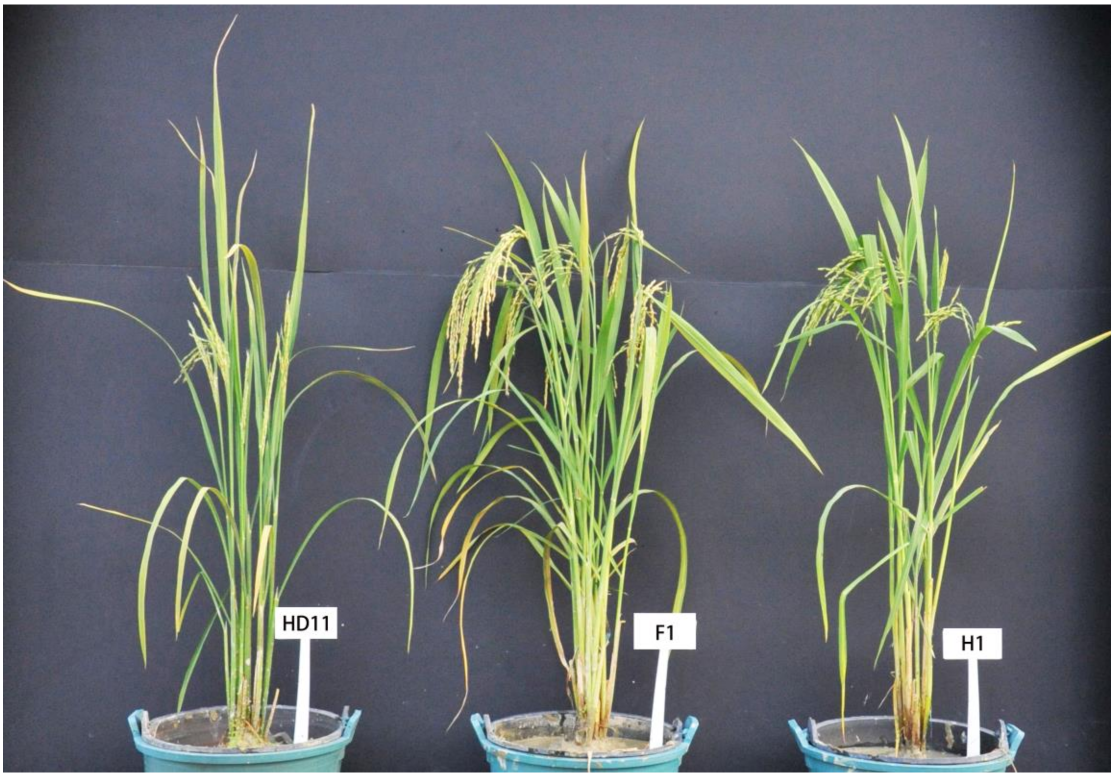


**Figure S1.** Plant morphology of neo-tetraploid hybrid rice (HD11×H1).

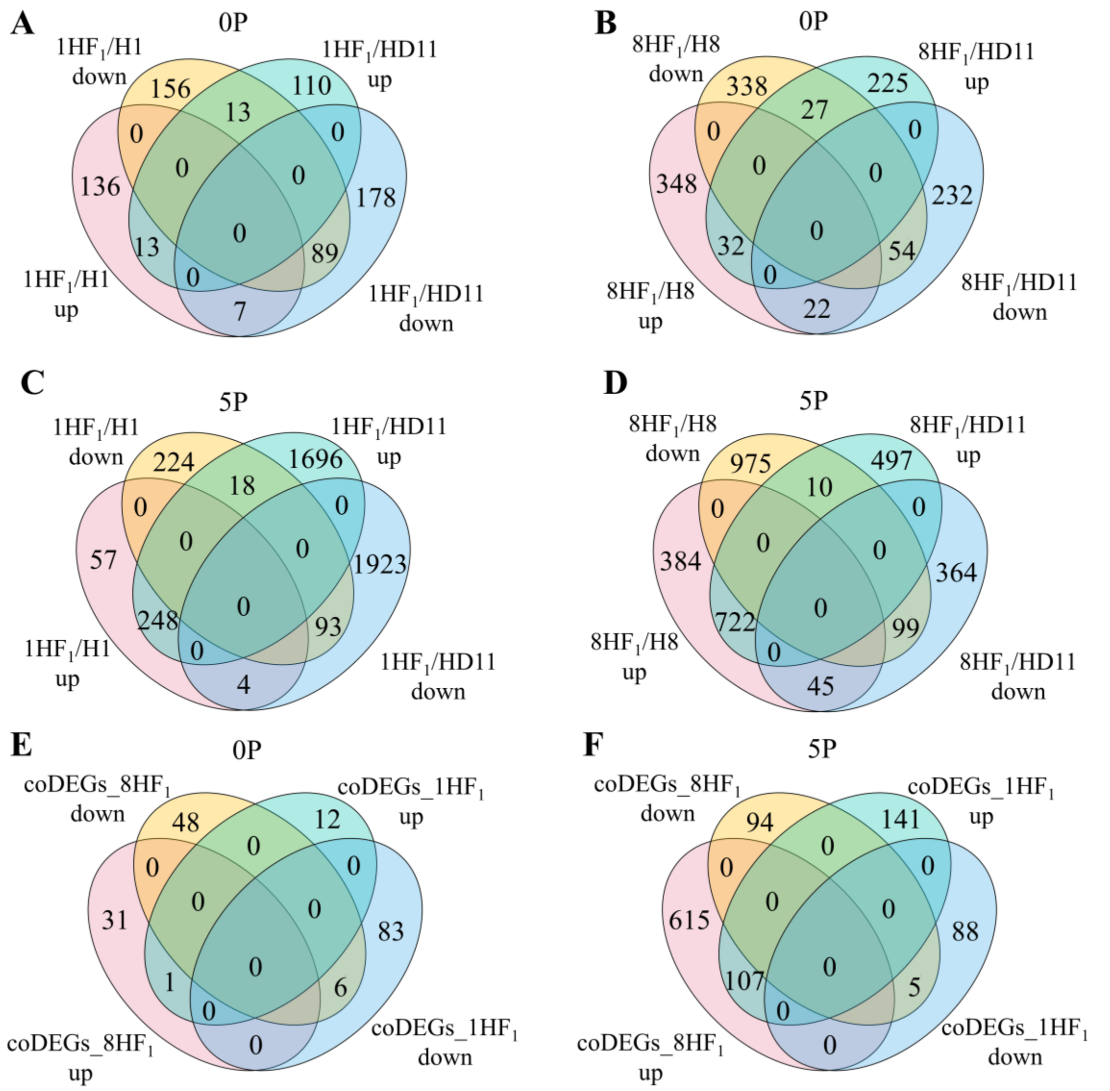


**Figure S2.** Identification of common differentially expressed genes of tetraploid hybrids.

(A) Identification of coDEGs in $1HF_1$ during 0P relative to H1 and HD11; (B) Identification of coDEGs in $8HF_1$ during 0P relative to H8 and HD11; (C) Identification of coDEGs in $1HF_1$ during 5P relative to H1 and HD11; (D) Identification of coDEGs in $8HF_1$ during 5P relative to H8 and HD11; (E) Identification of overlapped coDEGs in $1HF_1$ and $8HF_1$ during 0P; (F) Identification of overlapped coDEGs in $1HF_1$ and $8HF_1$ during 5P.

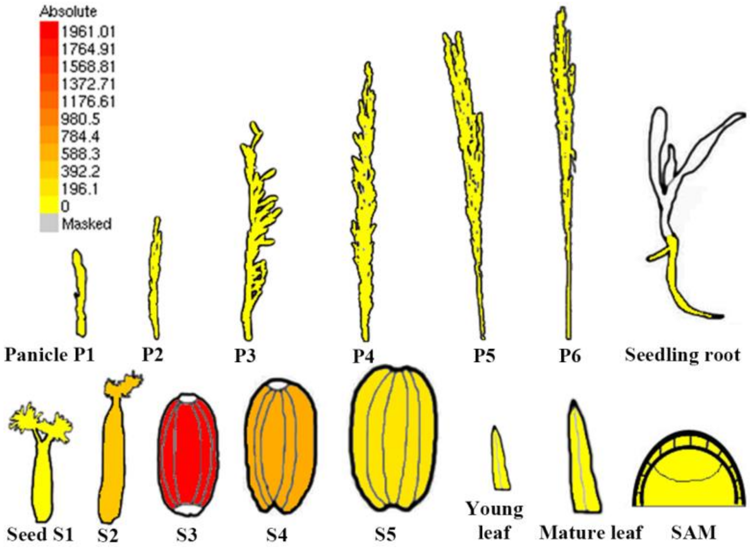


**Figure S3.** Expression pattern of *OsFl3* via eFP.

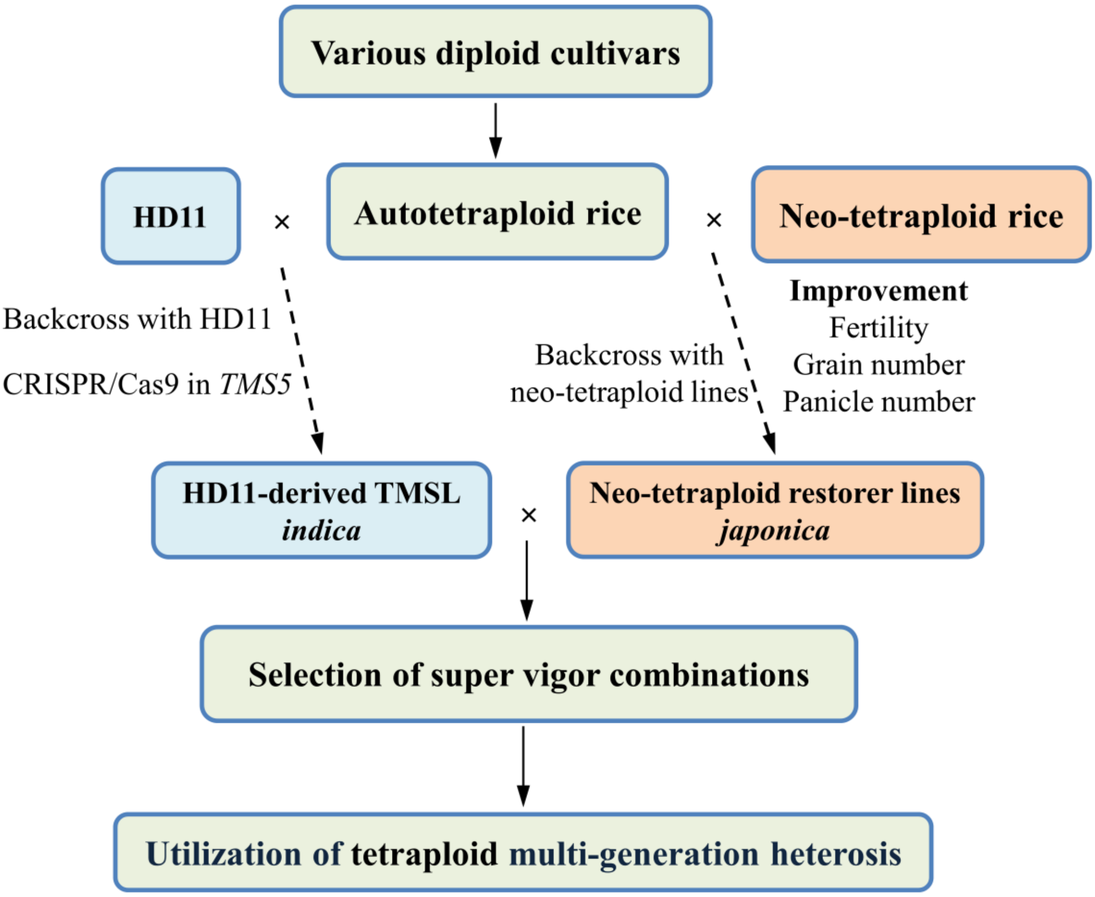


**Figure S4.** Key strategy for future intersubspecific tetraploid hybrid rice breeding.
